# Emotions affect the recognition of hand gestures

**DOI:** 10.3389/fnhum.2013.00906

**Published:** 2013-12-26

**Authors:** Carmelo M. Vicario, Anica Newman

**Affiliations:** School of Psychology, University of QueenslandBrisbane, Australia

**Keywords:** facial expressions, anger, happiness, pro-sociality, antisociality, hand posture

## Abstract

The body is closely tied to the processing of social and emotional information. The purpose of this study was to determine whether a relationship between emotions and social attitudes conveyed through gestures exists. Thus, we tested the effect of pro-social (i.e., happy face) and anti-social (i.e., angry face) emotional primes on the ability to detect socially relevant hand postures (i.e., pictures depicting an open/closed hand). In particular, participants were required to establish, as quickly as possible, if the test stimulus (i.e., a hand posture) was the same or different, compared to the reference stimulus (i.e., a hand posture) previously displayed in the computer screen. Results show that facial primes, displayed between the reference and the test stimuli, influence the recognition of hand postures, according to the social attitude implicitly related to the stimulus. We found that perception of pro-social (i.e., happy face) primes resulted in slower RTs in detecting the open hand posture as compared to the closed hand posture. Vice-versa, perception of the anti-social (i.e., angry face) prime resulted in slower RTs in detecting the closed hand posture compared to the open hand posture. These results suggest that the social attitude implicitly conveyed by the displayed stimuli might represent the conceptual link between emotions and gestures.

## Introduction

The body is closely tied to the processing of social and emotional information. Embodied cognition theories posit that knowledge is grounded in the brain's modal systems for perception, action, and affect (Candidi et al., 2010; Vicario et al., 2013). These systems are automatically engaged during online conceptual processing, thus allowing the re-enactment of modality-specific patterns of activity similar to those called into play during the actual experience of perception, action, and emotion (Barsalou et al., 2003; Barsalou, 2008).

Several versions of embodied cognition have been proposed in the last 20 years (for a discussion, see Wilson, 2002). A common aspect emphasized by embodied cognition theories is the simulation of experience in modality-specific systems. Examples include (Glenberg's 1997) theory of memory, Barsalou (1999) theory of perceptual symbol systems, and Damasio's (1994) theory of emotion. The main idea underlying all theories is that cognitive representations and operations are fundamentally grounded in their physical context (Niedenthal et al., 2005). For example, Reed and Farah (1995) asked a group of participants to judge whether two human figures depicted the same posture. The results showed that participants were better at detecting changes in the arm position of a visually presented figure while using their upper limb to generate a response and better at detecting changes in the figure's legs while moving their own legs to generate a response.

Interesting insights in support of the embodied cognition theories have also been provided in a study on attitudes, conceived by Darwin (1872/1904) as a collection of motor behaviors—especially postures—that convey an organism's affective response toward an object. For example, Wells and Petty (1980) instructed a group of participants to nod their heads vertically, or to shake their heads horizontally, while wearing headphones. While performing these movements, participants heard either a disagreeable or an agreeable message about a university-related topic. In a subsequent phase, they rated the degree to which they agreed with the message. The results showed that the participants' head movements modulated their judgments. Specifically, participants who nodded their heads while hearing the message judged it to be more favorable than participants who shook their heads.

Furthermore, embodiment is also critically involved in the representation of emotions. Niedenthal et al. (2001) demonstrated that facial mimicry plays a causal role in the processing of emotional expressions. Participants watched one facial expression morph into another and had to detect when the expression changed. Some participants were free to mimic the expressions, while others were prevented from mimicking by holding a pencil laterally between their lips and teeth. Consistent with the embodiment hypothesis, participants free to mimic the expressions detected the change in emotional expression more efficiently than did participants who were prevented from mimicking the expressions. This evidence (see Niedenthal et al., 2005 for other works on this argument) suggests that feedback from facial mimicry is important in a perceiver's ability to process emotional expressions.

Mimicry has been recently shown to relate to social attitudes. For example, Leighton et al. (2010) investigated whether social attitudes have a direct and specific effect on mimicry. To address this, a group of participants was primed with pro-social, neutral or anti-social words in a scrambled sentence task. They were then tested for mimicry using a stimulus-response compatibility task which required the execution of a pre-specified movement (e.g., opening their hand) on presentation of a compatible (open) or incompatible (close) hand movement. Results showed that pro-social priming produced a larger automatic imitation effect than anti-social priming, indicating that the relationship between mimicry and social attitudes is bidirectional, and that social attitudes have a direct and specific effect on the tendency to imitate behavior without intention or conscious awareness.

All the works discussed above suggest a relationship between social attitudes and the processing of emotions. In fact, an emotional expression is informative not only about the emotional state of a person, but also a signal of its affiliative intention (Hess et al., 2000). Accordingly, it was suggested that individuals who show happy expressions are perceived as highly affiliative, whereas individuals who show anger are perceived as highly non-affiliative, especially when the expresser is male (Knutson, 1996; Hess et al., 2000).

In consideration of this suggestion, in the current research we addressed the hypothesis that pro-social (i.e., happy) vs. anti-social (i.e., angry) facial expressions influence the recognition of the social attitudes implicitly conveyed by a particular hand posture (i.e., closed hand posture: anti-social attitude; open hand posture: pro-social attitude). In fact, as described by Givens (2008), uplifted palms (i.e., open hand) suggest a vulnerable or non-aggressive pose that appeals to listeners as allies rather than as rivals or foes. Moreover, Shaver et al. (1987) found that fist clenching (i.e., closed hand) is involved in the anger prototype.

Therefore, we used a same/different task (Farell, 1985), which is a behavioral paradigm classically used for testing the effect of task irrelevant stimuli (e.g., visual primes) on participants' performance. In fact, the time taken to make a same/different judgment can be a particularly useful measurement as it can be used to isolate the mental processes underlying a phenomenon of interest (Sternberg, 1969).

We predict that the “angry” prime (which reflects an anti-social attitude) selectively interferes with the reaction times (RTs) in detecting pictures depicting a closed hand posture (i.e., anti-social attitude) compared to open hand postures; vice-versa, we expect that the “happy” prime (which reflects a pro-social attitude) selectively interferes with RTs in detecting pictures depicting an open hand posture (i.e., pro-social attitude) compared to a closed hand posture. This prediction was made according to studies suggesting some relationship between social attitudes, emotion and embodiment (see Niedenthal et al., 2005 for a review).

In consideration of the paradigm used to explore our research goal (i.e., same/different task, Farell, 1985), we expect to detect performance interference (i.e., slower RTs) rather than performance facilitation (i.e., faster RTs), as predicted according to the results by Leighton et al. (2010). In fact, several previous works (Pratto and John, 1991; MacKay et al., 2004; Most et al., 2005; Ihssen et al., 2007) have shown that rapidly presented emotional pictures (as in the current study) interfere with performance in detecting other stimuli, probably because this type of presentation captures attentional resources. A recent study clarifies the mechanism behind the attentional interference reported in association with the involvement of emotional stimuli (Hodsoll et al., 2011). These authors showed, in five separate experiments that both positive (i.e., happy) and negative (i.e., fearful and angry) facial expressions interfere with RTs when the emotional stimulus was irrelevant for execution of the task (like in our study). Importantly, the RT interference was only reported when the emotional stimulus was irrelevant to the execution of the task, as in our study. Moreover, the same/different task adopted in our study implies the involvement of working memory processes, since participants were required to retrieve the reference stimulus to make the comparison. Accordingly, there is evidence of emotions having an “interference” effect on working memory (WM) processes. For example, the study by Kensinger and Corkin (2003) indicated that negative faces slow down responses in a non-verbal WM task. Unfortunately they did not use happy faces. Interestingly, this effect was only found with facial stimuli (not with emotional words), suggesting that the performance interference is specific for facial stimuli.

## Methods

### Participants

Thirteen consenting healthy participants with an average age of 22.71 ± 3.17 years, 4 male, were recruited from the University of Queensland (Australia). All were right-handed, had normal or correct-to-normal vision, and were proficient in the English language. Participants were all naïve to the purpose of the experiment. The experiment was performed in accordance with the ethical standards laid down in the 1964 Declaration of Helsinki.

### Stimuli and procedure

**Figure 1:**
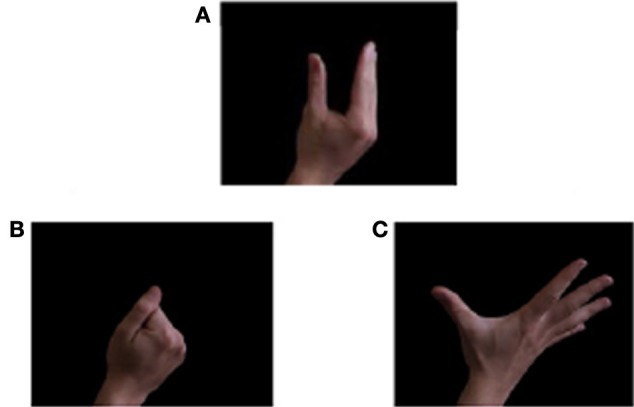
**Stimuli used for the experimental paradigm.** *Hand postures:* **(A)** Neutral hand posture; **(B)** Closed hand posture; **(C)** Open hand posture. Reprinted from Leighton et al. (2010).

Participants were positioned 50 cm from a Dell computer 21″ monitor configured at a refresh rate of 60 Hz. All the visual stimuli were presented in a single session which included the three hand postures (i.e., closed hand, open hand, neutral posture—control posture) used in the study by Leighton et al. (2010) (see Figure 1 for details) and three facial expressions (i.e., happy, angry, neutral expression—control priming).

**Figure 2:**
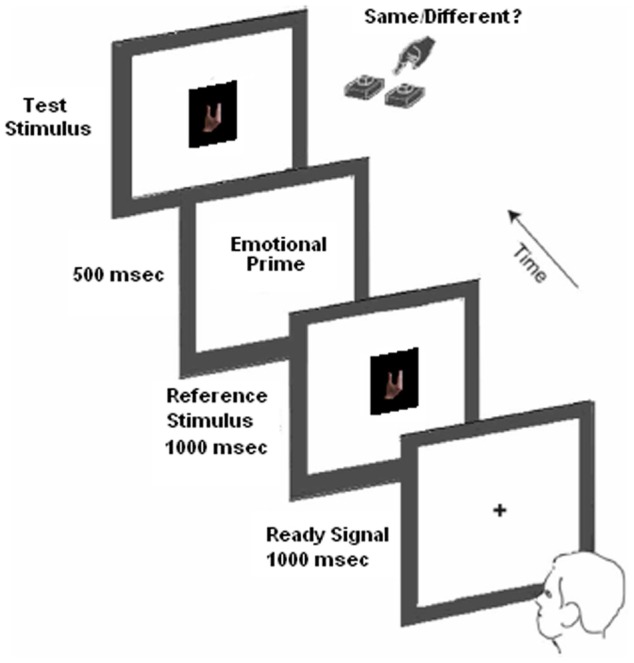
**Experimental procedure.** Example of typical event trials for the detection task. In the current task, participants were required to establish, as quickly as possible, whether the test stimulus was the same as or different to the reference stimulus.

The stimuli were presented in the center of the screen for a total of 270 trials (i.e., 90 trials for the “*same*” condition: 10 trials × 3 facial expressions × 3 hand postures; 180 trials for the “*different*” condition: 10 trials × 3 facial expressions × 6 combinations of the 3 hand postures). A typical trial sequence was presented as follows: First, the computer program displayed a ready signal (fixation cross) lasting 1000 ms. Next, a reference stimulus (i.e., one among the three hand postures) was presented for 1000 ms. Immediately after the reference stimulus disappeared, the computer program displayed a visual prime (i.e., one among the three face stimuli) lasting 500 ms. Finally, once the visual prime disappeared, the computer program displayed a test stimulus (i.e., one among the three facial stimuli. See Figure 2 for a typical trial sequence). By using one among two buttons of the keyboard (V and B-counterbalanced response across subjects) participants were required to establish, as quickly as possible, whether the test stimulus was the same as or different to the reference stimulus. Before testing, participants were required to complete 27 practice trials.

### Data analysis

Given the difference in the amount of conditions when the reference and test were the “*same*” compared to the conditions when the reference and test were “*different*,” we decided to collapse the data of the “*different*” conditions in order to obtain 3 categories of reference stimuli from the initial six combinations: (1) reference “*open*” test “*neutral*”; (2) reference “*open*” test “*closed.*” (3) reference “*closed*” test “*neutral*”; (4) reference “*closed*” test “*open*”; (5) reference “*neutral*” test “*open*”; (6) reference “*neutral*” test “*closed*.”

Before collapsing the data to obtain 3 categories, we performed 3 separate ANOVAs (one for each emotional prime) in order to verify whether the reference stimulus (*per se*) influenced the participants' performance. The results showed no significant difference among the 6 stimulus conditions for the neutral [*F*_(5, 60)_ = 0.73, *p* = 0.602, η*p*^2^ = 0.057, power = 0.245], as well as for the happy [*F*_(5, 60)_ = 2.03, *p* = 0.086, η*p*^2^ = 0.145, power = 0.639] and angry [*F*_(5, 60)_ = 1.78, *p* = 0.129, η*p*^2^ = 0.129, power = 0.574] facial expressions. Thus, we collapsed the data for all three emotional primes in order to obtain three “different” test stimulus categories: (1) *Neutral test stimulus category*: reference “*open*” test “*neutral*” with reference “*closed*” test “*neutral;* (2) *Open test stimulus category:* reference “*neutral*” test “*open*” with reference “*closed*” test “*open*”; (3) *Closed test stimulus category*: reference “*neutral*” test “*closed*” with reference “*open*” test “*closed.* A further analysis was performed on “same” condition trials, when a neutral prime was presented (i.e., neutral expression prime), to control for an effect of the reference/test stimulus compatibility on the participants' performance. No congruency effect was found for the three considered postures when a neutral expression prime was presented [*F*_(2, 24)_ = 1.33, *p* = 0.28, η*p*^2^ = 0.100, power = 0.260].

After collapsing the data of the different conditions, we normalized the RTs obtained for both angry and happy prime conditions by dividing them with those obtained for the neutral expression prime condition (i.e., baseline). Thus, normalized RTs were entered in a (2 × 2 × 3) factorial design with Stimulus (*same* and *different*), Facial expression (*anger* and *happiness*), Hand posture (*neutral, open, closed*), as main factors. *Post hoc* comparisons were performed with Fisher *post hoc* tests. For all tests, statistical significance was set at *p* < 0.05. Errors and false alarms were removed before performing the analysis. They were distributed as following: *Errors*: No expression “same”(1.02%), no expression “different” (0.85%); Anger “same” (1.2%), anger “different”(1.02%); Happiness “same” (1.20%), happiness “different (0.65%). *False alarm:* No expression “same”(1.05%), no expression “different” (0.54%); Anger “same” (1.4%), anger “different”(0.54%); Happiness “same” (1.08%), happiness “different (0.25%).

Data analysis was performed using Statistica software, version 8.0, StatSoft, Inc., Tulsa, OK, USA.

## Results

The repeated measures ANOVA revealed that there was no significant main effect for the Stimulus [*F*_(1, 12)_ = 0.001, *p* = 0.971, η*p*^2^ < 0.001, power = 0.050], Facial expression [*F*_(1, 12)_ = 0.842, *p* = 0.337, η*p*^2^ = 0.06, power = 0.135] and Hand posture [*F*_(2, 24)_ = 1.23, *p* = 0.309, η*p*^2^ = 0.093, power = 0.242] main factors. No significant results were found for the Stimulus × Facial expression [*F*_(1, 12)_ = 0.006, *p* = 0.939, η*p*^2^ < 0.001, power = 0.050], and Stimulus × Hand posture [*F*_(2, 24)_ = 0.105, *p* = 0.901, η*p*^2^ = 0.008, power = 0.064] interaction factors. However, a significant result for the Facial expression × Hand posture interaction factor was found [*F*_(2, 24)_ = 3.42, *p* = 0.049, η*p*^2^ = 0.221, power = 0.586]. In particular, we found that the RTs in detecting the closed hand posture were significantly slower compared to the open (*p* = 0.01) and the neutral (*p* = 0.03) hand postures, when presented with the angry prime. Analyses revealed a significant Stimulus × Facial expression × Hand posture interaction factor [*F*_(2, 24)_ = 6.73, *p* = 0.004, η*p*^2^ = 0.359, power = 0.878]. *Post hoc* comparisons showed a significant interaction exclusively for the “*same*” condition. In particular we found that RTs in detecting a closed hand posture were slower, compared to the RTs for both neutral (*p* = 0.04) and open (*p* = 0.01) hand postures, when the angry prime was presented. Vice versa, we found that the RTs in detecting both neutral (*p* = 0.02) and open (*p* = 0.03) hand postures were significantly slower, compared to the RTs for the closed hand posture, when the happy prime was presented.

**Figure 3:**
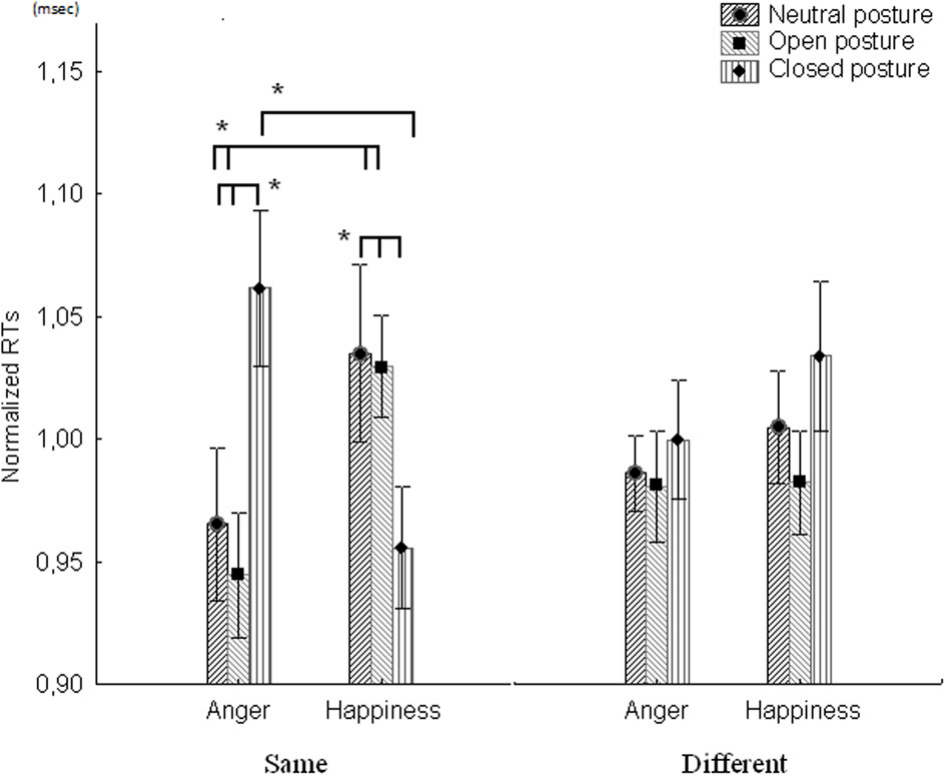
**Participants' performance in the same/different task.** The figure shows RTs (normalized by dividing them with the baseline condition- neutral facial expression) for both angry and happy primes when presented with Neutral, Open or Closed hand postures. The results show a significant difference for the “same” condition (i.e., same), and no significant difference for the “different” condition (i.e., different). ^*^Denotes *P-*values < 0.05. Vertical bars indicate standard errors of the mean.

Similar results were found for each single posture by comparing RTs when participants were presented with both emotional primes. In particular, we found that RTs for both neutral and open hand postures were slower in the happy prime condition (*p* = 0.045; *p* = 0.016, respectively) compared to the angry prime condition. Vice-versa, RTs for detecting the closed hand posture were slower in the angry prime condition (*p* = 0.003) compared to the happy prime condition. No significant differences were reported for the “*different*” conditions (see Figure 3 for details).

A further analysis was conducted to examine the accuracy of responses (Error %). A significant main effect for the Stimulus factor was detected [*F*_(1, 12)_ = 20.02, *p* < 0.001, η*p*^2^ = 0.627, power = 0.984]. In particular we found a lower accuracy in detecting same hand postures (*M* = 10.7% ± 1.53) with respect to different hand postures (*M* = 4.1% ± 0.65). We also detected a significant difference in accuracy of responses for Facial expressions [*F*_(2, 24)_ = 4.18, *p* = 0.027, η*p*^2^ = 0.258, power = 0.680]. In particular we found a higher error when the angry face was presented (*M* = 8.9% ± 1.19) as compared to both neutral (*M* = 6.7% ± 1.08, *p* = 0.031) and happy (*M* = 6.4% ± 0.95, *p* = 0.012) faces.

No other significant main effects were detected: Hand posture [*F*_(2, 24)_ = 0.45, *p* = 0.642, η*p*^2^ = 0.036, power = 0.114]. Likewise, no significant interaction effects were found: Stimulus × Facial expression [*F*_(2, 24)_ = 0.61, *p* = 0.547, η*p*^2^ = 0.049, power = 0.141], Stimulus × Hand posture [*F*_(2, 24)_ = 0.48, *p* = 0.619, η*p*^2^ = 0.039, power = 0.120], Facial expression × Hand posture [*F*_(4, 48)_ = 1.34, *p* = 0.268, η*p*^2^ = 0.100, power = 0.386] and Stimulus × Facial expression × Hand posture [*F*_(4, 48)_ = 1.09, *p* = 0.372, η*p*^2^ = 0.083, power = 0.316].

## Discussion

It was recently suggested that embodied simulation mediates our capacity to share the meaning of actions, intentions, feelings, and emotions with others, thus grounding our identification with and connectedness to others (Gallese, 2009).

In the current research we sought to investigate the existence of a relationship between emotion and embodiment. In particular, we were interested in testing the existence of a relationship between pro-social vs. anti-social facial expressions (i.e., happiness vs. anger) and pro-social vs. anti-social hand postures (i.e., open hand vs. closed hand postures). Thus, a same/different paradigm was used to investigate whether the RTs in recognizing a particular hand posture would have been affected by a particular emotional prime, depending on its social meaning.

Several studies have shown that exposure to faces expressing emotions (i.e., happiness or anger) affect facial mimicry (see Hess and Fischer, 2013 for a recent review). On the other hand, it has been shown that facial mimicry might be influenced by social attitudes. For example, when people watch a funny movie with friends, they laugh more than if they see the same movie alone (Jakobs et al., 1999); Moreover, there is evidence that facial expressions can affect the recognition of social attitudes. For example, a person showing happiness is typically perceived as having affiliative intentions, whereas a person showing anger or disgust is not (Hess et al., 2000).

Similarly to this research which demonstrates a link between emotion and social attitude, the results provided by the “same” condition of our study show that the presentation of happy facial primes resulted in slower RTs in detecting the open hand posture compared to the closed hand posture. Vice-versa, presentation of the anger facial prime slowed down RTs in detecting the closed hand posture compared to the open hand posture. We also found a pattern of results similar to those documented for the open hand posture while detecting the neutral hand posture. A possible suggestion for explaining this result is that the social meaning associated with the neutral posture was similar (i.e., pro-social) to that associated with the open hand posture. This is a likely interpretation, considering that the stimulus used for the neutral prime was a partially open hand. However, this suggestion remains to be verified since our participants were not asked to rate the grade of social attitude (pro-social vs. anti-social) associated with the three hand pictures.

On the other hand, no difference was reported for the “different” condition. Possible arguments for explaining this difference between the “same” and the “different” conditions can call into question (1) the higher number of trials (*n* = 180) for the “different” condition compared to the “same” condition (*n* = 90). This could have caused some habituation effect in the “different” condition that could have reduced the expected effect; (2) the higher number of combinations (i.e., 6 different trial conditions) which increases the perceptual variability (compared to the 3 different trial of the “same” condition).

One hypothesis regarding the application of theories of embodied cognition to emotion is that the perception of emotional meaning involves the embodiment of the implied emotion (Adolphs, 2002). Thus, it has been suggested that decoding these signals is accompanied by unconscious imitation as the perception of an individual's facial expression induces a corresponding reaction in the observer's facial muscles (Dimberg et al., 2000).

On the other hand, our study provides evidence of a direct link between emotion and embodiment, which extends beyond the unconscious imitation of the displayed facial expression. In fact, we found that the exposure to facial expressions affects the recognition of hand pictures, according to the social attitude implicitly related to the stimulus posture. This suggests that perceiving facial expressions might automatically pre-activate the expectation for social (pro vs. anti-social) outcomes which in turn affects the recognition of a social attitude implicitly conveyed through the hand gesture.

Our result provides new insights into the emotion/embodiment issue as it shows the existence of a particular relationship between emotions and gestures. In particular, it suggests that the “social attitude” might represent the link between gestures and emotions.

Our study bears limitations such as the limited number of participants and the absence of data about the degree to which each of the three hand postures are perceived to communicate pro-social and anti-social attitudes. This notwithstanding, we believe it creates the rationale for more extensive investigation of the emotion-embodiment issue.

Future works devoted to explore this issue might wish to investigate whether: (1) visual primes associated with a social attitude (i.e., pictures depicting open vs. closed hands) affect the recognition of pro-social vs. anti-social facial expressions (i.e., happiness vs. anger); (2) facial expressions such as those used in the current study affect hand mimicry, depending on the social attitude associated with the displayed posture; (3) mood manipulation (i.e., happiness) influences the recognition of socially relevant gestures. Moreover, to explore the soundness and the generalizability of these effects, it would be interesting to replicate this experiment through the use of different paradigms (i.e., go/no go task).

### Conflict of interest statement

The authors declare that the research was conducted in the absence of any commercial or financial relationships that could be construed as a potential conflict of interest.
